# Beyond the Hospital Walls: Integrating Clinical Wisdom and Global Equity Into the Circular Healthcare Paradigm – A Response to Recent Commentaries

**DOI:** 10.34172/ijhpm.10027

**Published:** 2026-06-20

**Authors:** Sandra C. Buttigieg, Ana Luísa Soares, Bartosz Bak, Sonya McFadden, Ciara Hughes, Patricia McClure, Jose Guilherme Couto, Isabel Bravo

**Affiliations:** ^1^Department of Health Systems Management and Leadership, Faculty of Health Sciences, University of Malta, Msida, Malta.; ^2^Medical Physics Service, Portuguese Oncology Institute of Porto, Porto, Portugal.; ^3^Radiotherapy Department II, Greater Poland Cancer Center, Poznan, Poland.; ^4^Faculty of Life and Health Sciences, Ulster University, Newtownabbey, UK.; ^5^Radiography Department, Faculty of Health Sciences, University of Malta, Msida, Malta.; ^6^Medical Physics and Radiobiology Group, Research Center (IPOP), Portuguese Oncology Institute of Porto, Porto, Portugal.

## Introduction: Toward a Collective Vision for Regenerative Healthcare

 The publication of our foundational analysis, “A Review of the Applicability of Current Green Practices in Healthcare Facilities,” has catalyzed a sophisticated dialogue that transcends operational efficiency.^1^ This commentary is a collective insight that fundamentally shifts our strategic focus from mere green practices, often paying lip service to the term, to a systemic regenerative ethos, thereby aligning with the Lancet Commission’s assertion that the climate crisis represents the greatest global health opportunity of the 21st century.^2^ It is a response to the six commentaries that are offered by Gan & Kim, Wu & Liang, Weimann, Zanobini et al, von Eiff & von Eiff, and Esmaeili, which constitute a rigorous international scholarly peer review.^3-8^ Our purpose here is to synthesize these diverse perspectives into a cohesive roadmap for future health policy, bridging the gap between theoretical frameworks and actionable realities of healthcare leadership, so as to achieve a revolutionary shift to an environmentally friendly health sector.^9^ Indeed, we must evolve from superficial and tactical greening of facilities toward a profound clinical transformation whereby sustainability and high-quality care are inextricably linked. The strategic management of circular healthcare provides the necessary synthesis to align operational efficiency with clinical excellence.^7^ Strategic management serves as the vital bridge between facility-level greening initiatives and profound clinical transformation, ensuring that sustainability becomes a core component of organizational identity.

## The Clinical-Environmental Link: Addressing Overdiagnosis and Low-Value Care

 The new frontier of healthcare sustainability is located at the intersection of clinical practice and ecological weight.^3^ In our initial review, we established that healthcare decisions are not environmentally neutral. Nevertheless, as Gan and Kim, and Wu and Liang correctly argue, the most significant driver of the sector’s carbon footprint is the delivery of care itself.^3,4^ Unnecessary medical interventions, redundant diagnostic imaging, and overprescribing are not only clinical failures, but they are primary environmental pollutants.^10,11^ While the 3Rs (Reduce, Reuse, Recycle) of the circular economy provide a baseline, our most potent strategic tool is the first R, which is Reduce. This is achieved through robust disease prevention and strict adherence to clinical guidelines to eliminate no-value care.

 Navigating the clinical-environmental link requires an expert understanding of complex ethical trade-offs.^3^ The mandate to optimize resources must be balanced against clinical efficacy to ensure patient outcomes are never compromised.^7^ Three examples include prostate cancer screening, breast cancer screening and structural incentives. For prostate cancerscreening,adhering to guidelines is crucial to prevent overdiagnosis in older men with significant comorbidities, thereby avoiding interventions with high environmental and human costs.^12^ In breast cancer screening, systematic screening for women aged 50 and older demonstrates significant mortality reduction, justifying resource expenditure through clear, high-value clinical results.^13^ With regard to structural incentives, we must acknowledge, as Gan and Kim note, that clinical decisions are often hampered by structural limitations and incentive systems that drive overdiagnosis, which is increasing the carbon footprint.^14,15^ Addressing these requires a systemic shift in how we value health literacy.

## The Human Dimension: Organizational Health Literacy as a Sustainability Driver

 Operationalizing a circular economy model requires more than technological interventions. It requires a shift in human behaviour which is the crux of any sustainable system. Zanobini et al provide a critical missing link by introducing organizational health literacy (OHL).^6^ OHL is the mechanism through which systems make health information accessible and actionable, and it serves as a direct enabler of clinical waste reduction. By bridging the gap between clinician limitations and patient expectations, OHL reduces the demand for low-value services. Specifically, OHL drives sustainability through four evidence-based pathways. The first is avoiding unnecessary utilization and educating patients on self-management and preventive care. Studies highlight that adequate health literacy leads to fewer emergency department visits.^16^ The second is streamlining care processes through shared decision-making that prevents redundant procedures. Research by Saini et al suggests that comprehensible clinical information and decision aids can prevent up to 20% of elective procedures.^11^ The third is enhancing medication adherence, which effectively means that better adherence reduces the carbon-intensive waste of unconsumed pharmaceuticals and prevents avoidable hospitalizations. The fourth is enabling low-carbon care by investing and preparing patients for telemedicine and safe home-based care. Patel et al found that telemedicine consultations save between 20 to 100 kg of CO_2_ emissions per visit by avoiding patient and staff travel.^17^

## A Global Perspective: Moving Beyond Eurocentrism to Equity

 To be truly transformative, the circular healthcare paradigm must move beyond its current Eurocentric focus.^5^ Edda Weimann highlights a critical limitation in existing literature, namely the relative absence of contexts from low- and middle-income countries (LMICs).^5^ These regions face distinct barriers, including weak policy frameworks and the absence of holistic recycling chains. Furthermore, the global nature of the biomedical manufacturing sector, which Esmaeili identifies as contributing 3.7% to environmental impacts through biomedical material and device manufacturing, disproportionately affects vulnerable populations in these regions.^8^

 In LMICs, the 3Rs must be reimagined as a tool for resilience and equity. Rather than the perpetual acquisition of new, single-use items, a focus on restoration and reutilisation can create local employment opportunities in economically disadvantaged settings.^5^ By prioritizing vulnerable populations, including children and older adults, who are most affected by microplastic and toxic waste pollution, we can move toward a globally inclusive vision of circular healthcare.^5^ This shift toward context-specific solutions in LMICs necessitates more standardized metrics to prove their long-term value and effectiveness.

## Refining the Toolkit: Life Cycle Assessment and Systemic Metrics

 To justify the significant investment required for a circular transition, policy-makers require rigorous data that moves beyond tactical adjustments. Wu and Liang propose the life cycle assessment as the gold standard for this task.^4^ An life cycle assessment provides a cradle-to-grave analysis, measuring a product’s impact from raw material extraction to end-of-life disposal. This is essential for determining when a reusable device, which incurs sterilization and energy costs, is truly superior to a disposable alternative.^4^

 From a pragmatic leadership perspective, we must utilize tools like environmentally extended input-output analysis. This is particularly useful when a complete life cycle inventory of all goods within a hospital system is not realistic, as it links financial data with emission factors to estimate systemic impacts. We must also prioritize clinically oriented functional units, such as the carbon cost per one magnetic resonance imaging scan, to ensure fair comparisons. While we explore novel technologies like artificial intelligence (AI)-driven energy management, we should prioritize the immediate, proven impact of telemedicine and digital health in decarbonizing our systems.^5^

## Conclusion: Toward a Regenerative Ethos

 The evolution of healthcare must lead us toward a proactive stance of actively healing the planet. This transition aligns with the One Health model and the World Health Organization’s (WHO’s) expanded mandate to support sustainable societies. We are moving from a state of *doing no harm* to a sophisticated, regenerative approach to health systems management and leadership.

 We call upon hospital administrators and researchers to move beyond siloed initiatives and adopt multifaceted, ethically-grounded frameworks. This includes, as Gan and Kim^3^ highlight: the San-Q framework, which utilizes six domains with dual metrics for implementation completion and significance, and the Seventy-Two Shades model, representing a paradigmatic shift toward multifaceted sustainability. By merging clinical wisdom with environmental ethics, we can fulfill our dual mandate: to restore our patients and to restore the biosphere they inhabit.

## Disclosure of artificial intelligence (AI) use

 Not applicable.

## Ethical issues

 Not applicable.

## Conflicts of interest

 Authors declare that they have no conflicts of interest. Authors have participated in a SAFE EUROPE project under the Erasmus+ Sector Skill Alliances programme [grant agreement: 2018e2993/001-001]. This project finished in 2021.
